# Enhanced depth imaging optical coherence tomography features of two types of Vogt–Koyanagi–Harada disease: fuzzy or lost pattern of the choroidal vasculature is of diagnostic value

**DOI:** 10.3389/fmed.2024.1339609

**Published:** 2024-04-24

**Authors:** Xinshu Liu, Shuling Wang, Yan An, Hao Zhang

**Affiliations:** ^1^Department of Ophthalmology, The Fourth People's Hospital of Shenyang, China Medical University, Shenyang, Liaoning, China; ^2^School of Business Administration, Shenyang Pharmaceutical University, Shenyang, Liaoning, China

**Keywords:** exudative retinal detachment, fuzzy or lost pattern of the choroidal vasculature, optical coherence tomography, optic disc swelling, Vogt-Koyanagi-Harada disease

## Abstract

**Objective:**

This study aimed to compare enhanced depth imaging optical coherence tomography (EDI-OCT) features of exudative retinal detachment (ERD) type and optic disc (OD) swelling type Vogt–Koyanagi–Harada (VKH) disease.

**Methods:**

Hospitalized VKH patients were retrospectively reviewed and classified into the ERD type and the OD swelling type. The EDI-OCT features were then analyzed.

**Results:**

The study included 32 ERD type and 15 OD swelling type VKH patients at the acute uveitis stage. The interval between the onset of ocular symptoms and the start of treatment in OD swelling type VKH disease was significantly longer compared to the ERD type (*p* < 0.001). A fuzzy or lost pattern of the choroidal vasculature was observed in 100% of VKH patients of both types. Moreover, high frequencies (greater than or equal to 50%) of fluctuations in the internal limiting membrane, interdigitation zone disruption, ERD, retinal pigment epithelium (RPE) folds, and ellipsoid zone disruption were observed in both types. Patients with OD swelling type VKH disease exhibited higher frequencies of OD swelling and hyperreflective substances above the RPE (*p* < 0.001 and *p* = 0.003, respectively), with lower frequencies of ERD and bacillary layer detachment (*p* = 0.012 and *p* < 0.001, respectively). At the convalescence stage, changes in the EDI-OCT images of 10 ERD type and 5 OD swelling type VKH patients were analyzed. The frequencies of the OCT features decreased with similar trends in both types of VKH disease.

**Conclusion:**

Although ERD type and OD swelling type VKH disease have their own unique characteristics, they share common EDI-OCT features. The Fuzzy or lost pattern of the choroidal vasculature that indicates choroidal inflammation may serve as a diagnostic aid for VKH disease, especially for the OD swelling type and the early-stage ERD type.

## 1 Introduction

Vogt–Koyanagi–Harada (VKH) disease is a multisystemic autoimmune disorder that mediated by T cells acting against antigens found on melanocytes. It is characterized by bilateral granulomatous panuveitis and associated with central nervous system, auditory, and integumentary involvement (1). The most characteristic manifestation of panuveitis is exudative retinal detachment (ERD) (2). However, in some patients, ERD is not remarkable, and optic disc (OD) swelling is the more prominent fundus finding (3). Okunuki et al. (3) reported the clinical features of two types of VKH disease, ERD and OD swelling, and highlighted the differences in disease course and treatment response. Nevertheless, OD swelling type VKH disease, which needs to be differentiated from other conditions, such as optic neuritis, elevated intracranial pressure, and other autoimmune inflammatory conditions and infections, still tends to be misdiagnosed (4–6).

Optical coherence tomography (OCT) provides *in vivo* images of the retina and the choroid and is widely used in evaluating and diagnosing fundus diseases. OCT studies primarily focused on the topographic features of the retina and the choroid in VKH disease. Gupta et al. (7) reported the presence of retinal pigment epithelium (RPE) striations in OCT images of acute VKH patients. Furthermore, Agarwal et al. (8) found that bacillary layer detachment (BLD) is a common finding in OCT images of patients with acute VKH disease. Enhanced depth imaging OCT (EDI-OCT) provides more information about the choroid. In Agarwal et al.'s (9) study, the EDI-OCT-derived choroidal vascularity index had a statistically significant reduction over time in VKH patients. Using the newly invented optical coherence tomography angiography (OCTA), Liang et al. (10) found that the choriocapillary vascular density decreased in VKH patients compared with normal controls. Despite prior researches, no research comparing the OCT features of ERD type and OD swelling type VKH disease has been reported. In the current study, EDI-OCT images of two types of VKH disease at acute uveitis and convalescence stages were analyzed to offer additional clues to the diagnosis and evaluation of these two types of VKH disease, especially the OD swelling type.

## 2 Subjects and methods

### 2.1 Subjects

We retrospectively reviewed the medical records of consecutively hospitalized VKH patients in the Fourth People's Hospital of Shenyang between June 2017 and August 2020. This study was approved by the Institutional Review Board of the Fourth People's Hospital of Shenyang and adhered to the tenets of the Declaration of Helsinki. Patients with acute VKH disease, who had not received prior treatment, were included. VKH disease was diagnosed in accordance with the revised criteria of the International Committee on Nomenclature (11). The follow-up information was collected from patients who received regular follow-ups for at least 2 months. Patients with a history of other retinal and/or choroidal diseases, myopia >6 diopters, and opaque optical media were excluded. All patients received oral prednisone at an initial dose of 0.8–1.0 mg/kg of body weight daily, followed by a gradual tampering of the dose thereafter (1, 12). Additional oral immunomodulatory agents, topical steroids, and mydriatics were administered when necessary. VKH patients were divided into two groups based on whether they had clinically prominent ERD at presentation by Dr. Zhang. Patients with clinically evident ERD with or without OD swelling detected by indirect ophthalmoscopy were defined as having ERD type VKH disease, and patients with clinically evident OD hyperemia and swelling with no ERD detected by indirect ophthalmoscopy were defined as having OD swelling type VKH disease (3).

### 2.2 OCT examination

**Figure 1:**
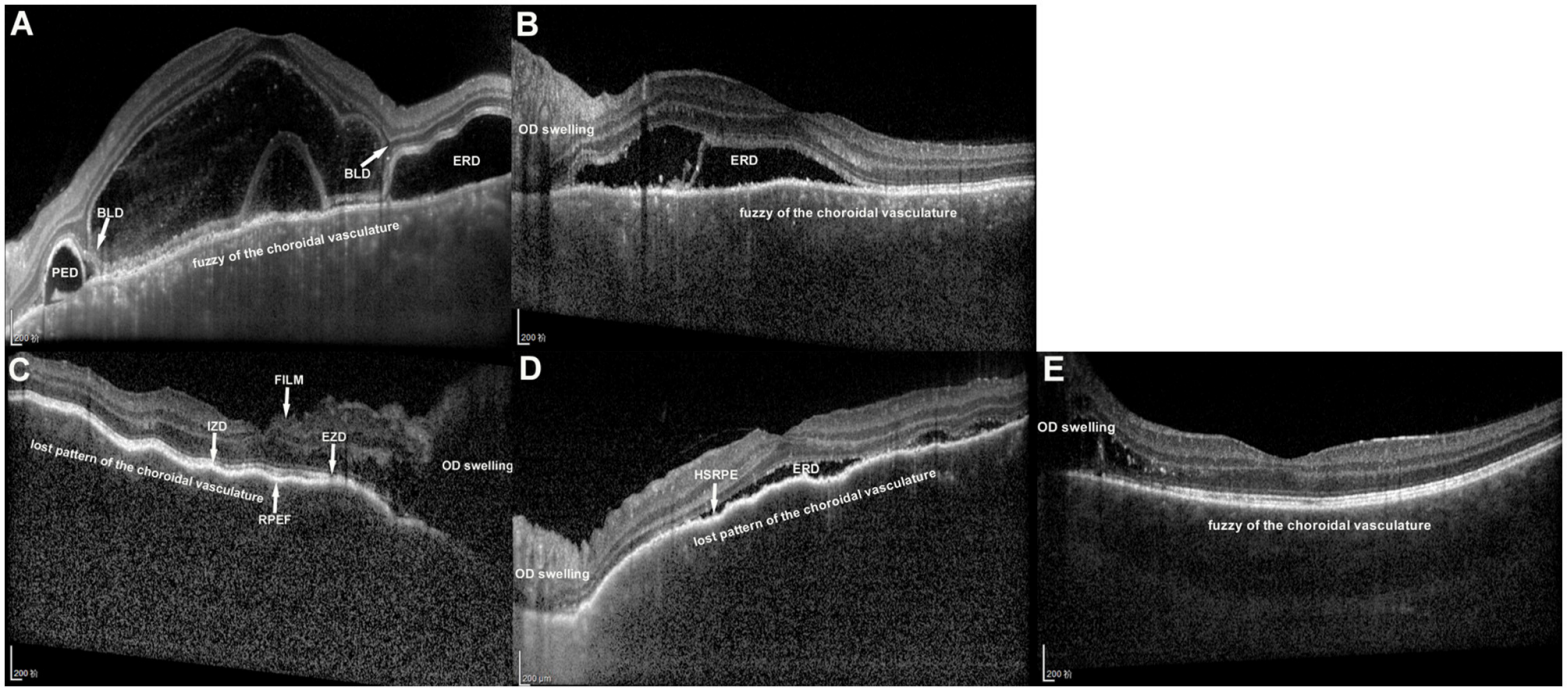
Enhanced depth imaging optical coherence tomography images of two types of Vogt–Koyanagi–Harada (VKH) disease at the acute uveitis stage. **(A)** The left eye of a patient with exudative retinal detachment (ERD) type VKH disease without optic disc (OD) swelling. ERD, bacillary layer detachment (BLD) and retinal pigment epithelium detachment (PED) were obvious. The pattern of the choroidal vasculature was fuzzy. **(B)** The left eye of a patient with ERD type VKH disease with OD swelling. The pattern of the choroidal vasculature was fuzzier than that in **(A)**. **(C)** The right eye of a patient with OD swelling type VKH disease with no ERD. OD swelling was obvious. Fluctuations in the internal limiting membrane (FILM), retinal pigment epithelium folds (RPEF), ellipsoid zone disruption (EZD) and interdigitation zone disruption (IZD) were displayed. The pattern of the choroidal vasculature was completely lost. **(D)** The left eye of a patient with OD swelling type VKH disease with shallow ERD. Hyperreflective substances above the RPE (HSRPE) was observed. The pattern of the choroidal vasculature was completely lost. **(E)** The left eye of a patient with OD swelling type VKH disease. Fuzzy of the choroidal vasculature was the only feature observed in addition to OD swelling.

OCT was performed using the Spectralis HRA+OCT system (Heidelberg Engineering, Heidelberg, Germany) fellow function at each visit. Raster scans with EDI were acquired at the macular region, with a minimum of 25 B-scans per volume scan of 30°*20°. Each B-scan was averaged over 9 frames, with a distance of 240 mm between consecutive scans. The OCT images at presentation and then at 1 week, 4 weeks, and 8 weeks after treatment initiation were independently reviewed by Dr. Liu and Dr. An. In cases of disagreement, Dr. Zhang made the final decision after a panel discussion. The following 10 OCT features of VKH patients were recorded in the current study: ERD, OD swelling, fluctuations in the internal limiting membrane (ILM), BLD, RPE folds, fuzzy or lost pattern of the choroidal vasculature, hyperreflective substances above RPE, ellipsoid zone (EZ) disruption, interdigitation zone (IZ) disruption, and RPE detachment (PED) (Figure 1). In cases of OD swelling type VKH disease, very mild ERD that was not observable during fundus examination was detectable by OCT. Fluctuations in the ILM refer to the loss of its smooth surface, resulting in fluctuating changes with at least 2 peaks or troughs (13). BLD was the break within myoids that isolates ellipsoids and outer segments from the remaining photoreceptor cell bodies (8, 14). RPE folds were the loss of the smooth surface of the RPE layer, resulting in various forms of protrusion. One peak or trough in the RPE layer was defined as an RPE fold (13).

### 2.3 Main outcome measures

The primary outcome of this study was EDI-OCT features of ERD type and OD swelling type VKH disease at the acute uveitis stage. We further observed the changes in these features at the convalescence stage.

### 2.4 Statistical analysis

Comparisons between the two types of VKH disease were conducted using SPSS 23.0 software for Windows (SPSS Inc., Chicago, IL, USA). Regarding demographic characteristics, the independent samples *t*-test was used for normal distribution continuous variables, the Mann–Whitney test was used for non-normal distribution continuous variables, and the chi-squared test was used for categorical variables. Generalized estimating equation was applied to accommodate the correlation between two eyes of the same person when comparing OCT features at the acute uveitis stage. *P* < 0.05 was considered statistically significant.

## 3 Results

This study included 47 VKH patients (94 eyes) presented at the acute uveitis stage, with 15 (30 eyes) of them receiving regular follow-ups for 2 months. Of the patients included, 32 patients (64 eyes) were classified as having ERD type VKH disease, while 15 patients (30 eyes) were classified as having OD swelling type VKH disease. The male percentages of the ERD type and the OD swelling type were 50.0% and 53.3%, respectively (*P* = 0.831). The average age at the onset of ocular symptoms in patients with the OD swelling type (49.2 ± 12.6 years) was higher than that in patients with the ERD type (42.5 ± 13.5 years); however, the difference was not statistically significant (*P* = 0.112). The interval between the onset of ocular symptoms and the start of treatment in the OD swelling type [12(9, 20) days] was significantly longer than that in the ERD type [7(4, 13) days] (*P* < 0.001). The demographic characteristics are presented in Table 1.

**Table 1:** Demographic characteristics of two types of Vogt-Koyanagi-Harada disease.

	**ERD type**	**OD swelling type**	***P***
Number of patients	32	15	NA
Number of eyes	64	30	NA
Male percentage	50.0%	53.3%	0.831^a^
Average age (years)	42.5 ± 13.5	49.2 ± 12.6	0.112^b^
Interval between the onset of ocular symptoms and the start of treatment (days)	7(4, 13)	12(9, 20)	**< 0.001** ^**c**^

ERD, exudative retinal detachment; OD, optic disc; ^a^chi square test, ^b^independent-samples t-test, ^c^Mann–Whitney test. Bold value was statistically significant.

Detailed information of EDI-OCT features of the two types of VKH disease at the acute uveitis stage is presented and compared in Table 2. OCT features with a frequency of 50% or higher were first evaluated. For ERD type VKH patients, the following frequencies of OCT features were observed: ERD (100%), fluctuations in the ILM (100%), fuzzy or lost pattern of the choroidal vasculature (100%), IZ disruption (100%), RPE folds (87.5%), EZ disruption (70.3%), and BLD (65.6%). For OD swelling type VKH patients, the following frequencies of OCT features were observed: OD swelling (100%), fuzzy or lost pattern of the choroidal vasculature (100%), fluctuations in the ILM (93.3%), IZ disruption (93.3%), RPE folds (86.7%), ERD (76.7%), EZ disruption (53.3%), and hyperreflective substances above RPE (50%). Moreover, we observed a low frequency of PED (10.9% in the ERD type and 10% in the OD swelling type) in both types. Notably, up to 60% of the OCT features showed a high level of consistency between the two types of VKH disease, except for the following statistically significant differences: In cases of the OD swelling type, the frequencies of OD swelling and hyperreflective substances above the RPE were higher than that in the ERD type (100% vs. 23.4% and 50% vs. 10.9%; *p* < 0.001 and *p* = 0.003); while the frequencies of ERD and BLD were lower than that in the ERD type (76.7% vs. 100% and 0 vs. 65.6%; *p* = 0.012 and *p* < 0.001).

**Table 2:** Enhanced depth imaging optical coherence tomography features of two types of Vogt-Koyanagi-Harada disease at the acute uveitis stage.

**Features**	**ERD type (*n =* 64)**	**OD swelling type (*n =* 30)**	***P***
ERD	64 (100.0%)	23 (76.7%)	**0.012**
OD swelling	15 (23.4%)	30 (100.0%)	**< 0.001**
Fluctuations in the ILM	64 (100.0%)	28 (93.3%)	0.301
Bacillary layer detachment	42 (65.6%)	0 (0)	**< 0.001**
RPE folds	56 (87.5%)	26 (86.7%)	0.937
Fuzzy or lost pattern of the choroidal vasculature	64 (100.0%)	30 (100.0%)	NA
Hyperreflective substances above RPE	7 (10.9%)	15 (50.0%)	**0.003**
EZ disruption	45 (70.3%)	16 (53.3%)	0.251
IZ disruption	64 (100.0%)	28 (93.3%)	0.301
PED	7 (10.9%)	3 (10.0%)	0.894

ERD, exudative retinal detachment; OD, optic disc; ILM, internal limiting membrane; RPE, retinal pigment epithelium; EZ, ellipsoid zone; IZ, interdigitation zone; PED, RPE detachment. Bold values were statistically significant.

The frequencies of all EDI-OCT features presented downward trends on different levels after treatment initiation in both types. More specifically, ERD and RPE folds resolved within 8 weeks and 4 weeks, respectively, in both types. In the ERD type, OD swelling subsided within 8 weeks, and fluctuations in the ILM and BLD subsided within 4 weeks; In the OD swelling type, fuzzy or lost pattern of the choroidal vasculature and EZ disruption regressed within 8 weeks. The frequencies of the fuzzy or lost pattern of the choroidal vasculature, hyperreflective substances above RPE, and EZ disruption in the ERD type and the OD swelling type, fluctuations in the ILM and hyperreflective substances above RPE in the OD swelling type were all below 40% within 8 weeks. IZ disruption and PED only improved within 8 weeks in a few cases of both types.

## 4 Discussion

In the present study, the OD swelling type VKH patients got longer interval between the onset of ocular symptoms and the start of treatment. OD swelling type VKH disease shares some common EDI-OCT features with ERD type VKH disease at the acute uveitis stage. The fuzzy or lost pattern of the choroidal vasculature was observed in 100% of the patients of both types of VKH, and fluctuations in the ILM, IZ disruption, ERD, RPE folds and EZ disruption were observed in at least 50% of the patients. Meanwhile, patients with OD swelling type VKH disease had higher frequencies of OD swelling and hyperreflective substances above the RPE, with lower frequencies of ERD and BLD. A good response to systematic anti-inflammatory treatment was observed in all OCT features of both types.

In our study and Okunuki et al.'s (3) study, the interval between the onset of ocular symptoms and the start of treatment in OD swelling type VKH disease was significantly longer than that in ERD type VKH disease. Two possible reasons may explain the treatment delay. On the one hand, compared to the dramatic decreased visual acuity due to abrupt progression of ERD in the ERD type, visual impairment in OD swelling type VKH disease was less severe due to no or subclinical ERD (detectable by OCT) at disease onset. Patients sought health care after a delay. On the other hand, the diagnosis of OD swelling type VKH may delay. In Le et al.'s (5) report, an OD swelling type VKH patient received her diagnosis after excluding other autoimmune, malignant, or infective etiologies following extensive investigations and a nearly 1 year period of observation. The treatment delay may be associated with chronic recurrent disease course and poor visual prognosis.

The fuzzy or lost pattern of the choroidal vasculature, the only choroidal OCT feature recorded and analyzed in the present study, was originally described as an indocyanine green angiography (ICGA) sign of VKH disease (15). Miyanaga et al. (15) observed a fuzzy or lost pattern of the choroidal vasculature in the intermediate-to-late phase of ICGA in all the initial acute VKH patients studied and presumed it to be an indication of diffuse inflammatory vasculopathy of choroidal vessels. We also observed this phenomenon in EDI-OCT images, and it was the only OCT feature observed in 100% of VKH patients of both types at the acute uveitis stage. During severe inflammation, the pattern of the choroidal vasculature was completely lost. With the remission of inflammation, the outline of the individual choroidal vessels gradually became clear. Lee et al. (16) observed a darkened type choroid morphology in EDI-OCT images of acute VKH patients. The darkened morphology was characterized as diffuse, homogenous, and hypo-reflective pattern with markedly decreased visibility of the large choroidal vessel layer (16), which is similar to our study's fuzzy or lost pattern of the choroidal vasculature. Although the fuzzy or lost pattern of the choroidal vasculature was observed in both ICGA and EDI-OCT images of acute VKH patients, we presumed different imaging principles. We suppose that, at the acute uveitis stage, edematous choroidal stroma and RPE cells caused by choroidal inflammation may block and scatter the near-infrared light reflected from the choroidal vasculature during OCT imaging, leading to a fuzzy or lost pattern of the choroidal vasculature. The fuzzy or lost pattern of the choroidal vasculature in EDI-OCT images, an indication of choroidal inflammation, is useful for the diagnosis of OD swelling type VKH disease, which tends to be misdiagnosed as optic neuritis or elevated intracranial pressure because of prominent OD swelling. It is also valuable for diagnosing very early-stage ERD type VKH disease, when the choroidal inflammation has not yet involved adjacent structures.

Fluctuations in the ILM, ERD, RPE folds, and OD swelling occur when choroidal inflammation progresses. Lin et al. (17) first observed and defined fluctuations in the ILM in OCT images of 34 out of 65 VKH eyes (52.3%). They also proposed local constriction caused by inflammatory cells infiltrating in the vitreous and diffuse, but uneven, edema of the retina and the choroid due to inflammation may be the cause (17). We also observed fluctuations in the ILM in more than 50% of the eyes of both types of VKH disease patients, and the frequencies of fluctuations in the ILM of two types of VKH showed no significant difference. In our study, the subclinical ERD detected by OCT at the acute uveitis stage of OD swelling type VKH disease was shallow and localized compared to the high and extensive ERD of the ERD type. Moreover, the frequency of ERD was observed to be lower in the OD swelling type. Gupta et al. described RPE folds, also known as RPE undulations, as peaks and troughs of the RPE layer in all 4 VKH patients (eight eyes) studied with 3-dimensional OCT (7). Hosoda et al. (18) hypothesized that the thick and deformed inflammatory choroid of VKH patients contributes to the formation of RPE folds. RPE folds were also common in our study, seen in more than 80% of eyes of both types of VKH disease patients, with the frequencies between the two types showing no significant difference. Fluctuations in the ILM, ERD, and RPE folds were common OCT features in both types of VKH disease, which may help in the diagnosis. The frequency of OD swelling at the acute uveitis stage of OD swelling type VKH disease was significantly higher than that of the ERD type. A previous study speculated that older age and crowded discs correlate with the occurrence of OD swelling (19). The average age of OD swelling type VKH disease was higher than that of the ERD type; however, it is not significant in the present study, and a larger sample size is needed to show statistical significance. Further studies on the choroidal circulation and disc morphology of OD swelling type VKH disease are warranted. These OCT features responded rapidly to systemic corticosteroid treatment in both types of VKH disease in our study. All of these features regressed within 8 weeks, except OD swelling and fluctuations in the ILM in the OD swelling type which may be due to severe OD hyperemia and swelling. Close monitoring of these OCT features would help in the evaluation of therapeutic efficacy.

OCT features related to tissue disorganization (BLD, EZ disruption, and IZ disruption) of VKH disease are discussed below. BLD, a common OCT feature of ERD type VKH disease, described as the cystoid space and membranous structure previously (20), was termed and defined by Mehta et al. (14) as the break within inner segment myoids that isolate ellipsoids and outer segments from the remaining photoreceptor cell bodies. Hydrostatic forces originating from choroidal inflammation and strengthening adherence at the IZ by the subretinal fibrin may both contribute to the split in the structural weakness myoid layer of VKH patients (8, 21). From Agarwal et al.'s (8) report, BLD was found in 112 of the 118 (94.9%) VKH eyes with serous retinal detachment at presentation, all of which resolved within 3.4 ± 1.3 days after intravenous methylprednisolone therapy (8). The percentage of BLD was 65.6% in ERD type VKH eyes at the acute uveitis stage in our study, and all regressed within 4 weeks after oral prednisone therapy. The percentage of BLD in the ERD type in the present study is lower than that in the former report (8), which may be due to different inclusion criteria. In the former report, only patients with multifocal serous retinal detachment were included (8), which may exclude less severe ERD type VKH patients. Based on the consensus of the International Nomenclature for Optical Coherence Tomography Panel, the outer three hyperreflective bands that were identified included the EZ, IZ, and RPE/Bruch's complex (22). Zhao et al. (13) observed the disappearance of the three-layer structure on OCT images of acute uveitis stage VKH patients. In our study, disruption of the EZ occurred in 70.3% of the eyes in the ERD type and 53.3% of the eyes in the OD swelling type, while disruption of the IZ occurred in 100% of the eyes in the ERD type and 93.3% of the eyes in the OD swelling type at the acute uveitis stage. We propose two explanations to help illustrate the occurrence of EZ and IZ disruption: outer segments and ellipsoids of photoreceptor cells injured in sequence by inflammation from the choroid and ellipsoids and outer segment degeneration after isolating from the remaining photoreceptor cell bodies in the BLD region. The EZ was gradually restored after systematic anti-inflammatory treatment. IZ restoration was slow (10% in the ERD type and 40% in the OD swelling type within 8 weeks), and the hyperreflective band of IZ may not be observed in some VKH patients until years after presentation based on our clinical experience.

Hyperreflective substances above the RPE may be constituted by degenerated photoreceptor outer segments, inflammatory exudation, and hyperplasia of RPE cells (23) after a period of disease onset. The frequency of hyperreflective substances above the RPE was higher in OD swelling type VKH disease than that in the ERD type at the acute uveitis stage. The longer interval between the onset of ocular symptoms and the start of treatment in OD swelling type than that in ERD type may be responsible. Degenerated photoreceptor outer segments and inflammatory exudation increase over time, and hyperplasia of RPE cells occurred at a certain time. Degenerated photoreceptor outer segments and inflammatory exudation are gradually resolved; however, hyperplasia of RPE cells remains (24), which may help explain why a small number of hyperreflective substances above the RPE still existed until the 8-week follow-up.

PED on OCT images is less common in VKH disease. In our study, it was observed in 7 out of 64 eyes (10.9%) and 3 out of 30 eyes (10%) at the acute uveitis stage of ERD type and OD swelling type VKH disease, respectively. Khochtali et al. (25) reported two acute VKH disease patients with bilateral PED. Although resolution of PED after systemic corticosteroids would suggest an underlying inflammatory mechanism, the exact process of PED is still unclear.

## 5 Conclusion

The current study compared EDI-OCT features of ERD type and OD swelling type VKH disease at acute uveitis and convalescence stages and explored the underlying physiopathological mechanisms. Although ERD type and OD swelling type VKH disease bear their own unique characteristics, they share common OCT features. Fuzzy or lost pattern of the choroidal vasculature indicates choroidal inflammation and was observed in all VKH patients at the acute uveitis stage. This may serve as a diagnostic aid for VKH disease, especially for the OD swelling type and the early-stage ERD type. All OCT features responded well to systematic anti-inflammatory treatment. Close monitoring of these OCT features would help in the evaluation of therapeutic efficacy.

## Data availability statement

The raw data supporting the conclusions of this article will be made available by the authors, without undue reservation.

## Ethics statement

The studies involving humans were approved by the Institutional Review Board of the Fourth People's Hospital of Shenyang. The studies were conducted in accordance with the local legislation and institutional requirements. Written informed consent for participation was not required from the participants or the participants' legal guardians/next of kin in accordance with the national legislation and institutional requirements.

## Author contributions

XL: Conceptualization, Data curation, Formal analysis, Funding acquisition, Investigation, Methodology, Resources, Software, Validation, Visualization, Writing – original draft, Writing – review & editing. SW: Supervision, Visualization, Writing – review & editing. YA: Data curation, Formal analysis, Investigation, Writing – original draft. HZ: Conceptualization, Investigation, Project administration, Supervision, Writing – review & editing.

## References

[1] Du L Kijlstra A Yang P. Vogt-Koyanagi-Harada disease: Novel insights into pathophysiology, diagnosis and treatment. Prog Retin Eye Res. (2016) 52:84–111. 10.1016/j.preteyeres.2016.02.002 26875727

[2] Herbort CP Tugal-Tutkun I Abu-El-Asrar A Gupta A Takeuchi M Fardeau C. Precise, simplified diagnostic criteria and optimised management of initial-onset Vogt-Koyanagi-Harada disease: an updated review. Eye. (2022) 36:29–43. 10.1038/s41433-021-01573-3 34145419 PMC8727674

[3] Okunuki Y Tsubota K Kezuka T Goto H. Differences in the clinical features of two types of Vogt-Koyanagi-Harada disease: serous retinal detachment and optic disc swelling. Jpn J Ophthalmol. (2015) 59:103–8. 10.1007/s10384-014-0367-8 25465197

[4] Nichani P Christakis PG Micieli JA. Headache and bilateral optic disc edema as the initial manifestation of Vogt-Koyanagi-Harada disease. J Neuroophthalmol. (2021) 41:e128–30. 10.1097/WNO.0000000000000917 32102009

[5] Le TA Simon S Gilhotra J Hissaria P. Vogt-Koyanagi-Harada syndrome presenting with bilateral optic disc swelling and leptomeningeal enhancement. BMJ Case Rep. (2019) 12:e229719. 10.1136/bcr-2019-229719 31118177 PMC6559813

[6] Yang HK Park KH Kim JS Hwang JM. Bilateral disc edema in a patient with Vogt-Koyanagi-Harada disease. Can J Ophthalmol. (2014) 49:e54–6. 10.1016/j.jcjo.2014.01.002 24767242

[7] Gupta V Gupta A Gupta P Sharma A. Spectral-domain cirrus optical coherence tomography of choroidal striations seen in the acute stage of Vogt-Koyanagi-Harada disease. Am J Ophthalmol. (2009) 147:148–53. 10.1016/j.ajo.2008.07.028 18834577

[8] Agarwal A Freund KB Kumar A Aggarwal K Sharma D Katoch D. Bacillary layer detachment in acute Vogt-Koyanagi-Harada disease: A novel swept-source optical coherence tomography analysis. Retina. (2021) 41:774–83. 10.1097/IAE.0000000000002914 32833410

[9] Agrawal R Li LK Nakhate V Khandelwal N Mahendradas P. Choroidal vascularity index in Vogt-Koyanagi-Harada disease: an EDI-OCT derived tool for monitoring disease progression. Transl Vis Sci Technol. (2016) 5:7. 10.1167/tvst.5.4.7 27525196 PMC4970799

[10] Liang A Jia S Gao F Han X Pei M Qu Y. Decrease of choriocapillary vascular density measured by optical coherence tomography angiography in Vogt-Koyanagi-Harada disease. Graefe's Arch Clin Exp Ophthalmol. (2021) 259:3395–404. 10.1007/s00417-021-05238-5 34216256 PMC8523392

[11] Read RW Holland GN Rao NA Tabbara KF Ohno S Arellanes-Garcia L. Revised diagnostic criteria for Vogt-Koyanagi-Harada disease: report of an international committee on nomenclature. Am J Ophthalmol. (2001) 131:647–52. 10.1016/s0002-9394(01)00925-4 11336942

[12] Yang P Ye Z Du L Zhou Q Qi J Liang L. Novel treatment regimen of Vogt-Koyanagi-Harada disease with a reduced dose of corticosteroids combined with immunosuppressive agents. Curr Eye Res. (2018) 43:254–61. 10.1080/02713683.2017.1383444 29111815

[13] Zhao GL Li RZ Pang YH Wang XQ Peng HJ Wei JF. Diagnostic function of 3D optical coherence tomography images in diagnosis of Vogt-Koyanagi-Harada Disease at acute uveitis stage. Med Sci Monit. (2018) 24:687–97. 10.12659/msm.905931 29396390 PMC5807914

[14] Mehta N Chong J Tsui E Duncan JL Curcio CA Freund KB. Presumed foveal bacillary layer detachment in a patient with toxoplasmosis chorioretinitis and pachychoroid disease. Retin Cases Brief Rep. (2021) 15:391–8. 10.1097/ICB.0000000000000817 30142112

[15] Miyanaga M Kawaguchi T Miyata K Horie S Mochizuki M Herbort CP. Indocyanine green angiography findings in initial acute pretreatment Vogt-Koyanagi-Harada disease in Japanese patients. Jpn J Ophthalmol. (2010) 54:377–82. 10.1007/s10384-010-0853-6 21052896

[16] Lee H Bae K Kang SW Woo SJ Ryoo NK Kim SJ. Morphologic characteristics of choroid in the major choroidal thickening diseases, studied by optical coherence tomography. PLoS ONE. (2016) 11:e147139. 10.1371/journal.pone.0147139 26766530 PMC4713229

[17] Lin D Chen W Zhang G Huang H Zhou Z Cen L. Comparison of the optical coherence tomographic characters between acute Vogt-Koyanagi-Harada disease and acute central serous chorioretinopathy. BMC Ophthalmol. (2014) 14:87. 24974016 10.1186/1471-2415-14-87 PMC4099160

[18] Hosoda Y Uji A Hangai M Morooka S Nishijima K Yoshimura N. Relationship between retinal lesions and inward choroidal bulging in Vogt-Koyanagi-Harada disease. Am J Ophthalmol. (2014) 157:1056–63. 10.1016/j.ajo.2014.01.015 24491415

[19] Nakao K Abematsu N Mizushima Y Sakamoto T. Optic disc swelling in Vogt-Koyanagi-Harada disease. Invest Ophth Vis Sci. (2012) 53:1917–22. 10.1167/iovs.11-8984 22408010

[20] Ishihara K Hangai M Kita M Yoshimura N. Acute Vogt–Koyanagi–Harada disease in enhanced spectral-domain optical coherence tomography. Ophthalmology. (2009) 116:1799–807. 10.1016/j.ophtha.2009.04.002 19643489

[21] Liakopoulos S Keane PA Ristau T Kirchhof B Walsh AC Sadda SR. Atypical outer retinal fluid accumulation in choroidal neovascularization: a novel OCT finding. Ophthal Surg Lasers Imag Retina. (2013) 44:S11–8. 10.3928/23258160-20131101-03 24220879

[22] Staurenghi G Sadda S Chakravarthy U Spaide RF. Proposed lexicon for anatomic landmarks in normal posterior segment spectral-domain optical coherence tomography. Ophthalmology. (2014) 121:1572–8. 10.1016/j.ophtha.2014.02.023 24755005

[23] Miura M Makita S Azuma S Yasuno Y Sugiyama S Mino T. Evaluation of retinal pigment epithelium layer change in Vogt-Koyanagi-Harada disease with multicontrast optical coherence tomography. Invest Ophth Vis Sci. (2019) 60:3352–62. 10.1167/iovs.19-27378 31917451

[24] Nakamura T Hayashi A Oiwake T. Long-term changes of retinal pigment epithelium in the eyes with Vogt-Koyanagi-Harada disease observed by adaptive optics imaging. Clin Ophthalmol. (2019) 13:927–33. 10.2147/OPTH.S199886 31213764 PMC6549749

[25] Khochtali S Ksiaa I Megzari K Khairallah M. Retinal pigment epithelium detachment in acute Vogt-Koyanagi-Harada disease: an unusual finding at presentation. Ocul Immunol Inflamm. (2019) 27:591–4. 10.1080/09273948.2018.1433304 29513616

